# Recurrent Deep Network Models for Clinical NLP Tasks: Use Case with Sentence Boundary Disambiguation

**DOI:** 10.3233/SHTI190211

**Published:** 2019-08-21

**Authors:** Benjamin C. Knoll, Elizabeth A. Lindemann, Arian L. Albert, Genevieve B. Melton, Serguei V.S. Pakhomov

**Affiliations:** a Institute for Health Informatics, University of Minnesota, Minneapolis, Minnesota, USA; b Department of Surgery, University of Minnesota, Minneapolis, Minnesota, USA; c College of Pharmacy, University of Minnesota, Minneapolis, Minnesota, USA

**Keywords:** Natural Language Processing, Machine Learning, Neural Networks (Computer)

## Abstract

Although a number of foundational natural language processing (NLP) tasks like text segmentation are considered a simple problem in the general English domain dominated by well-formed text, complexities of clinical documentation lead to poor performance of existing solutions designed for the general English domain. We present an alternative solution that relies on a convolutional neural network layer followed by a bidirectional long short-term memory layer (CNN-Bi-LSTM) for the task of sentence boundary disambiguation and describe an ensemble approach for domain adaptation using two training corpora. Implementations using the Keras neural-networks API are available at https://github.com/NLPIE/clinical-sentences.

## Introduction

In contrast to general English, clinical notes have significant differences in structure and content. For instance, clinical text often contains units of thought that fit the technical definition of sentences that are not terminated by the standard sentence boundary symbols or any symbols in many cases. Structures such as labels, section headers, text arranged in tables, and lists are examples of clinical text that do not follow general English rules for sentence termination. Furthermore, clinical text contains a disproportionately high number of acronyms, abbreviations, and ordinal numbers frequently decorated with punctuation symbols and containing variable capitalization. Segmentation errors caused by these ambiguities are magnified in downstream processing.

Previous research has shown that transfer learning in deep networks can improve generalization to tasks of related problems with small data sets [1]. Ensemble methods that engage in meta-learning through weighted voting models such as boosting, bagging, and stacking also reduce the generalization error over standard models [2]. We utilize transfer learning both in the use of word embeddings, and in our method for domain adaptation of models trained on one corpus to a different, but related corpus of clinical text.

Sentence boundary disambiguation (SBD), also known as sentence segmentation or sentence boundary detection, is a well-understood and explored problem in the domain of general English text. In well-formed general English text, most sentences are terminated by sentence boundary symbols. Ambiguities caused by acronyms, abbreviations, quotations, and ordinal numbers are handled by further rules, or by statistical methods such as maximum entropy classification of boundaries. Using these methods on general English text results in accuracy performance above 95% [3]. Relying on sentence boundary symbols for SBD on the entire text of a clinical document leads to errors in detecting non-terminated sentence boundaries [4]. Although deep learning is the prevailing approach for many machine learning problems, it remains underutilized in clinical applications, and the generalizability of clinical applications using current approaches is limited [5].

In this paper we report on applying deep neural network methods with the use case of sequence labeling to the SBD problem on the entire text of clinical notes with no preprocessing or cleaning. We show that an architecture consisting of word embeddings enriched with character information run through a bi-LSTM have high accuracy in detecting sentence boundaries. We also explore the generalization problem of using a trained model for SBD on previously unseen text using several implementations, including combining training data, resuming training with data from the new corpus, and a stacking method where the hidden layer results of two models trained separately on both corpora are summed before prediction using a shared prediction layer. We show that the stacking method has the lowest generalization error with 96% F1 score for beginning of sentence tags.

## Methods

### Sentence Segmentation

SBD is often the first step in solving any problem using natural language processing (NLP). Availability of sentence boundaries is necessary both for many general language tasks, such as part-of-speech tagging and parsing, and for domain-specific analytical tasks such as document classification. Errors in sentence detection tend to propagate to many other areas in a system making sentence accuracy critical for any downstream tasks in a text analysis system.

In Table 1, some examples of text from Fairview Medical Services notes where sentences are not terminated with sentence boundary symbols are shown.

There are several existing commonly used implementations of SBD that rely on or expect sentence boundary symbols and perform poorly in their absence. Stanford CoreNLP [6] provides a rule-based algorithm, which makes decisions based on the results of a tokenizer to disambiguate whether sentence boundary symbols indicate sentence splits. Natural Language Toolkit (NLTK) [7] implements SBD using a method that combines rules for sentence boundaries with an unsupervised algorithm for the detection of acronyms and abbreviations, a common source of errors in SBD [8]. The Apache OpenNLP toolkit [9] and Apache cTAKES [10] provide SBD based on the maximum entropy method described in Reynar, et. al [3]. Previous evaluations have looked at the performance of SBD and have noted the difficulty of the task in the domain of clinical notes and have noted the performance issues on non-terminated sentences [4].

Approaches to the NLP problem of sequence tagging are well-suited for the SBD problem—which can be expressed as a tagging task where words beginning sentences are tagged ‘B’ and words internal to sentences are tagged ‘I’. The architecture for sequence tagging involving recurrent neural networks (RNNs) has shown good results when applied to the tasks of part-of-speech (PoS) tagging and named entity recognition (NER) [11], and improvements were shown when character information is combined with word embeddings via a convolutional neural network (CNN) [12].

### Dataset

#### Source Corpora

We created two source corpora for the sentence detection task. The first dataset was drawn from the MIMIC-III (Medical Information Mart for Intensive Care) corpus [13], which is a de-identified corpus of notes associated with 40,000 intensive care unit patients at the Beth Israel Deaconess Medical Center between 2θ01 and 2012. Our MIMIC corpus consisted of 749 randomly sampled notes. A second, target dataset was drawn from the Fairview Health Services (FV) EHR system. We used a stratified sampling strategy, in which we created batches of 56 notes made up of 16 inpatient notes and 40 outpatient notes. The inpatient notes were selected proportional to the distribution of note type (Table 2) and the outpatient notes were selected proportional to the distribution of department (Table 3). A total of 952 notes from FV were used, 17 complete batches. MIMIC notes were in plaintext while FV notes were converted from RTF to plaintext using the BioMedICUS system [14]. The MIMIC corpus used in this study contains a total of 315,797 tokens, and the FV corpus contained 415,112 tokens.

#### Manual Annotation of Sentences

The manual annotation of sentences was performed in the BRAT Rapid Annotation Tool [15] by a pair of trained annotators. Annotators were instructed to label all complete thoughts, section headers, item labels, list items, and fragments using a “Sentence” annotation. For any data that was not groupable into sentence-like units (e.g., purely numeric tables, lists of laboratory data, lines of vital signs measures, and metadata tables such as those in header information), or for any other areas of text for which annotators had low confidence in their ability to correctly label sentences, annotators were instructed to use an “Unsure” annotation. After sentences were manually annotated, the documents were tokenized and converted to tagged sequences where ‘B’ was applied to the first token in every sentence, ‘I’ was applied to the rest of the tokens in the sentence, and ‘O’ was applied to all the tokens in the “Unsure” category.

#### Cross-validation structure

To evaluate generalization error, a cross-validation structure was used where 100% of the MIMIC data was used for cross-validation with 80% as a training split and 20% as a validation split; for the FV data 50% was used for cross-validation (again using an 80-20 training-validation split) and 50% was held out as an unseen test corpus. For architecture and hyper-parameter tuning, the MIMIC validation split was used. The FV cross-validation set was used for training models alone or augmenting MIMIC-trained models. During training, the validation data was used to provide validation loss as an estimation of generalization error to determine when the model has stopped improving and training could be halted.

### Model Architecture

**Figure 1 –:**
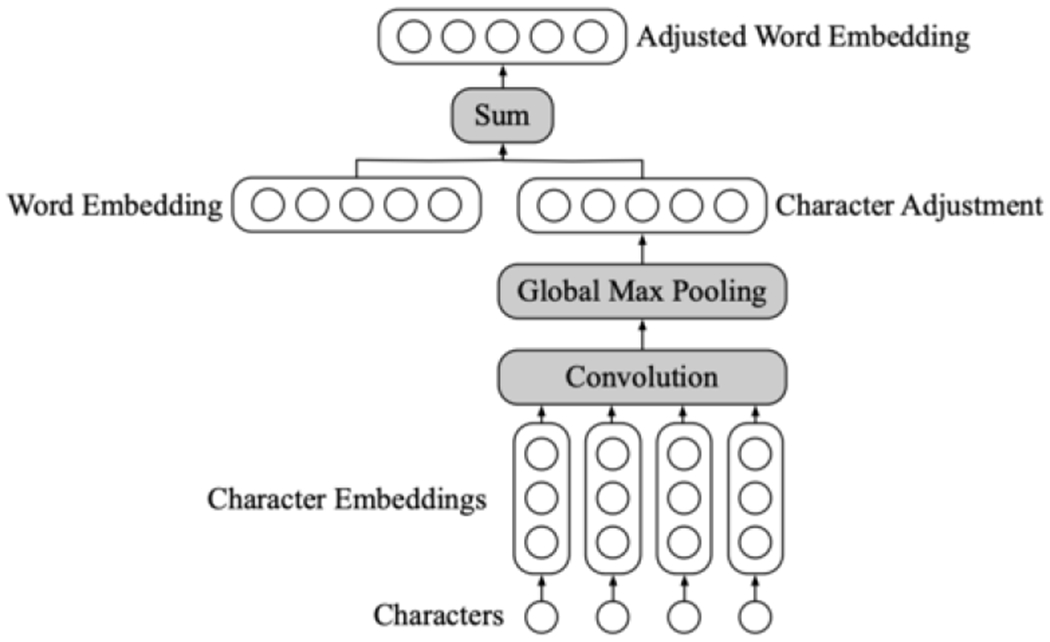
Word Representation Layer

Words were tokenized according to rules that split whenever any whitespace, any symbols, or any digits are encountered. Words were represented using a 300-dimension word embedding trained using the Facebook fastText software package [16] on the entire MIMIC-III corpus preprocessed to replace any symbols with spaces, to replace digits with their English names in separate words, i.e. “1.23” to “one two three”, and to lowercase all letters. These word embeddings are enriched by summing with the results of a convolutional neural network (CNN) on 30-dimension character embeddings which are learned during training on the SBD tagging task. The CNN is made up of one convolutional layer with 300 filters, each looking at the sequences of the embeddings of four characters, followed by global max pooling. The results of the CNN function as an adjustment vector to the original word vector for the sentence tagging task. The input of the character CNN is all characters of the word (including symbols and whitespace) along with a context of up to seven characters between the previous word and the word; and up to seven characters between the word and the next word. Special characters were inserted for the end of the previous word, the beginning of the next word, the beginning and end of the word, as well as the beginning and end of the document if those fell into the context. Using all the original characters, including whitespace, allows structural information about the document’s formatting to be used for SBD decisions. After the word representation is constructed the results are batch-normalized before passing to the next layer. The architecture of the word representation layer is shown in Figure 1.

**Figure 2 –:**
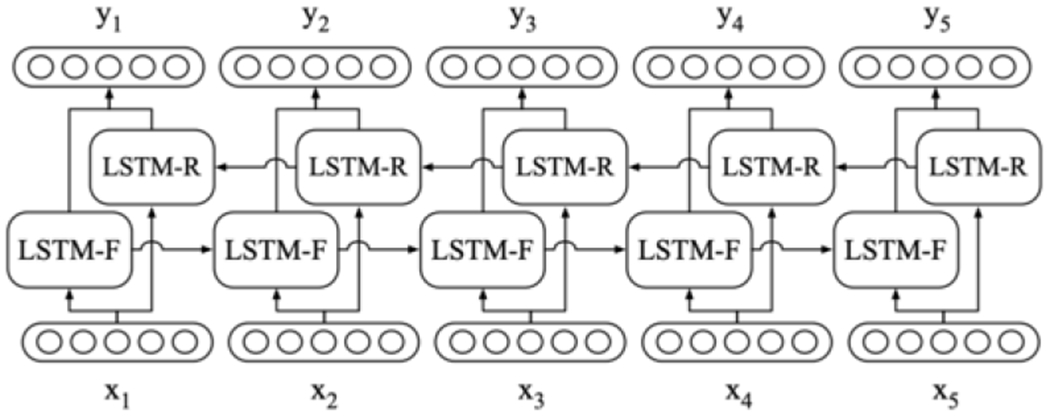
Bi-directional LSTM

To encode contextual word representations, we use a bidirectional long short-term memory (LSTM) layer. LSTM units are iteratively run on time-series data (sequences of words in the case of text), maintaining an internal cell state as it moves from one input to the next. LSTMs are optimized during training to learn what information is important to remember from previous inputs to the cell. A LSTM layer is parameterized by the number of LSTM units, each contributing one output dimension. In a bi-directional LSTM layer, the inputs are run both ways through the layer, with one set of LSTM units responsible for seeing the data in order and one set responsible for seeing the data in reverse. Dropout and recurrent dropout [17] were used to provide regularization of the learned weights and prevent overfitting. The results of the bi-LSTM layer are batch-normalized before being passed to the inference layer. In Figure 2, computation of a bi-LSTM on time series data is shown, each node labeled LSTM-F and LSTM-R is the same set of units at different points in the time series, and the lines drawn between nodes represent the propagation of internal memory states to the next item in the time series. The outputs from the forward and backward LSTMs are concatenated to a single contextual word representation, an embedding of the word and surrounding words.

**Figure 3 –:**
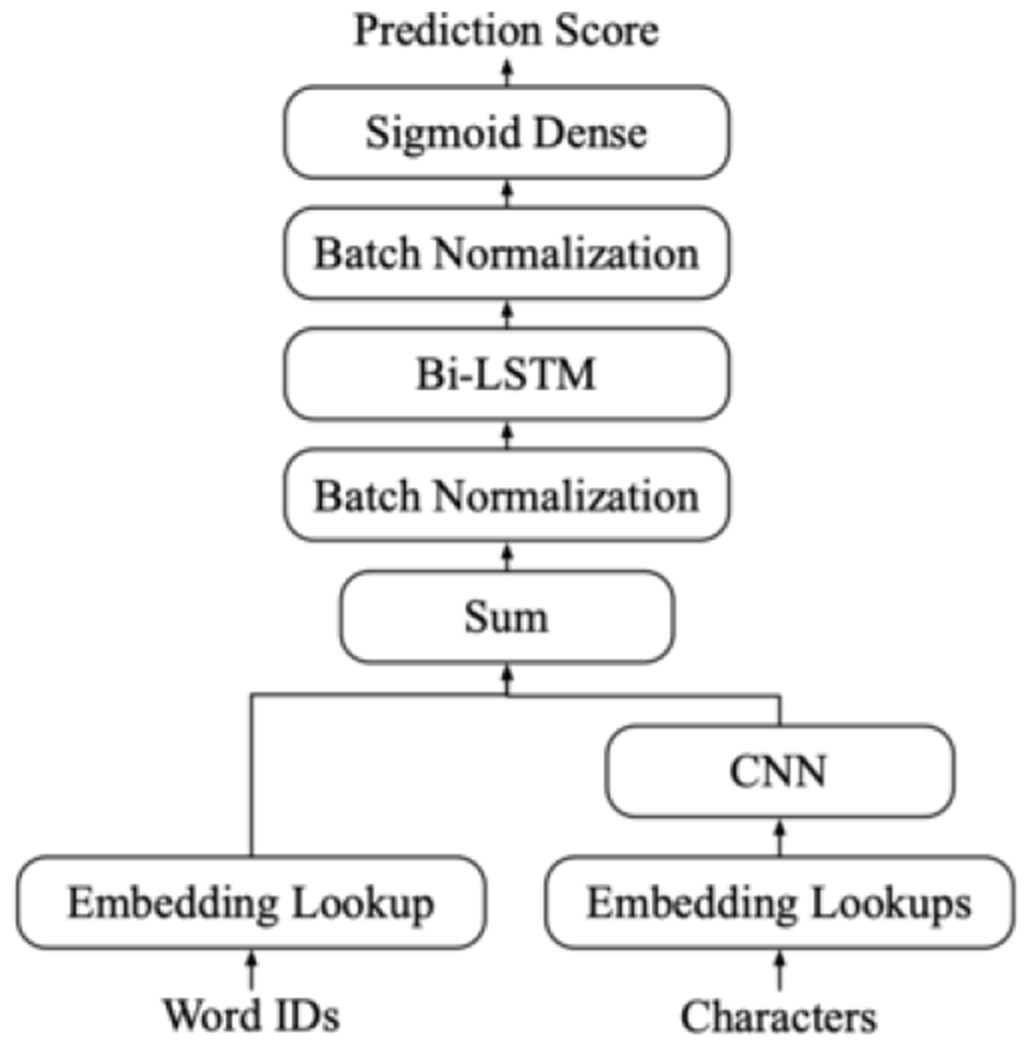
Complete Model Graph

After the bi-directional LSTM layer, a sigmoid-activated dense NN prediction layer is used on each contextual word embedding to output the log-probability that the word is the beginning of a sentence. Lasso or L1-norm regularization was used on the weights of the prediction layer to prevent overfitting. The complete graph of our architecture is shown in Figure 3.

#### Domain Adaptation

**Figure 4 –:**
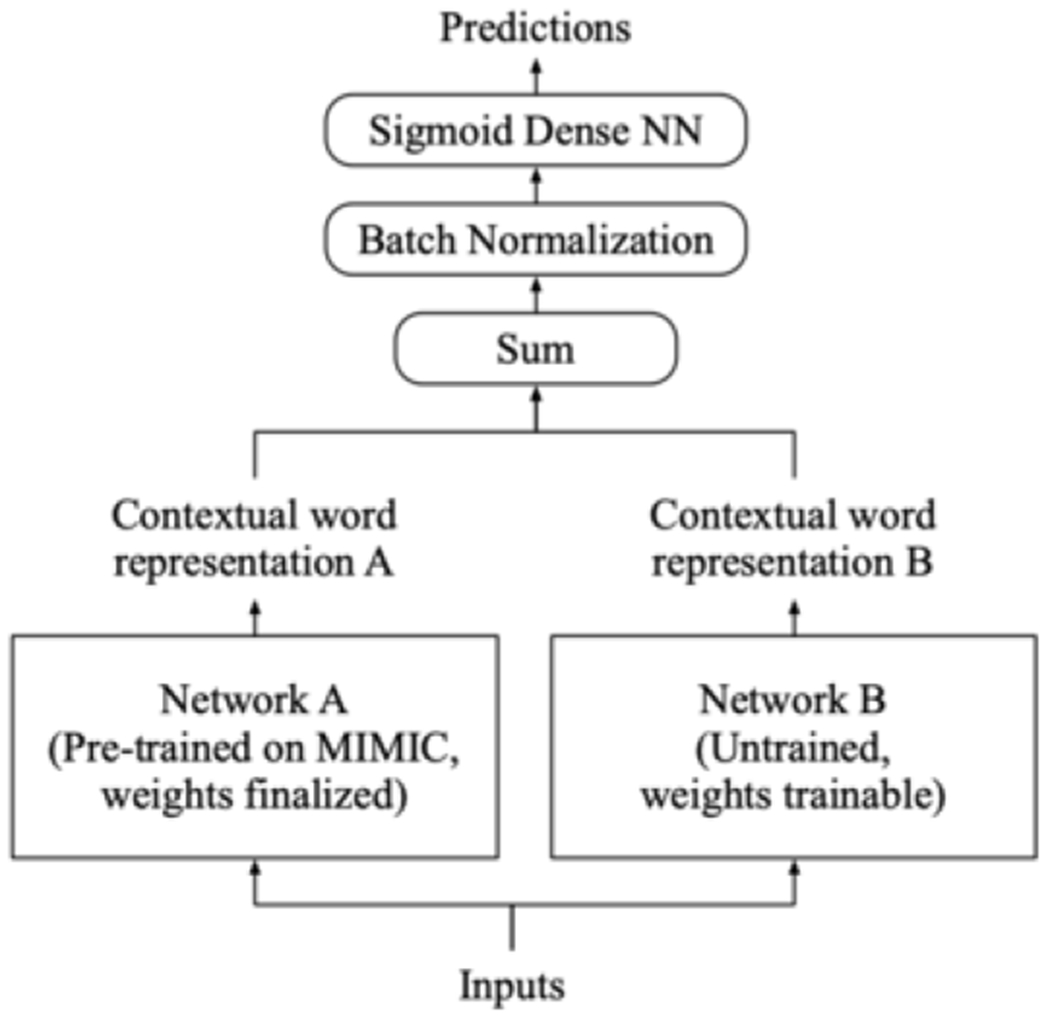
Ensemble of Two Networks

In addition to using models trained on each individual corpus, we evaluated three methods for domain adaptation of models trained on MIMIC to the FV hold-out test set. First, we looked at merging the cross-validation data from both corpora. Second, we looked at resuming training of the network trained on the MIMIC cross-validation data with the FV cross-validation data. Third, we looked at using an ensemble stacking method for transfer learning where the hidden-layer contextual word representations of the network trained on MIMIC were summed with the contextual word representations of a new network before the sigmoid dense NN prediction layer. In this architecture the output of the second network functions as corrections to the first network for the FV training data. This stacked network architecture is shown in Figure 4.

### Training

**Figure 5 –:**
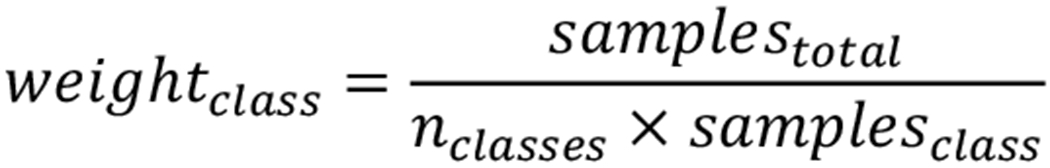
Weighting of classes

Based on the results of tuning using CV on the MIMIC corpus, we selected the gradient descent variant ADAM (Adaptive Moment Estimation) [18] as the optimizer of network weights. During training, only models that were improvements on validation loss were saved, and after 5 epochs with no improvement training was terminated. Binary cross-entropy loss was used for training, and loss values were weighted by the ratio between the target tag probability and an equal distribution of tags, shown in the equation in Figure 5. Mini-batching was used for training, sequences of 32 words were batched into groups of 32 for gradient optimization.

## Results

### Manually Annotated Corpora

On an overlap of 100 MIMIC notes annotated by both annotators, ignoring “Unsure” annotations, the Cohen’s kappa of Sentence annotations was computed as 0.957 using the irr library in R version 3.4.4. The agreement between annotators on “Unsure” annotations was 0.646. After conversion, on the subset of 100 notes labeled by both annotators Cohen’s kappa was 0.71 for all tags and 0.95 after excluding items labeled as ‘O’ by either annotator. Tables 4 and 5 describe the distribution of ‘B’, ‘I’, and ‘O’ tags after this conversion.

In both source corpora, sentences not terminated by sentence boundary symbols are highly prevalent. Section headers and text labels were common and often ended by the colon sentence boundary symbol. Table 6 shows the quantity of sentences terminated by each symbol.

### Evaluation of SBD Approaches

For our evaluation we ignored all tags that were labeled as ‘O’ both during training and during evaluation. Thus, the recall, precision, and F1 for ‘B’ and ‘I’ are symmetric, every false positive ‘B’ is a false negative ‘I’ and every false negative ‘B’ is a false positive ‘I’. We’ve reported only the ‘B’ scores as they are directly proportional to the overall accuracy of detected sentences. The best architecture and hyper-parameter tuned models from cross validation achieved 98.6% F1 on both ‘B’ and ‘I’ tags on the MIMIC validation set and 99.2% F1 on ‘B’ and ‘I’ tags in the FV validation set.

We evaluated our implementation of SBD against the 50% FV hold-out data set (476 notes). In addition to the architecture described above, we evaluated a maximum entropy / logistic regression classifier (listed as LR) using optimization of a sigmoid-activated dense NN layer on an input of 7 characters before, at the beginning, at the end, and following every word. This is an approach like, but not as tuned as individual implementations of maximum entropy SBD. The primary metrics used for evaluation were the precision, recall, and F1-score for the beginning of sentence class tag.

In addition to models trained on MIMIC and FV individually, we evaluated three methods to test generalizability against the FV test corpus. The models trained solely against one corpus are listed as “MIMIC” and “FV.” The results of a model trained on a both corpora’s cross-validation set combined are listed as “MIMIC+FV.” The continued training of one network is listed as “MIMIC then FV” and the ensemble model is listed as “Ensemble.” Results of these evaluations are show in Table 7 with best results in bold.

## Discussion

The complexity, grammatic idiosyncrasies, and domain variability of clinical text lead to significant hurdles in designing and training generalizable models for NLP tasks. This common challenge necessitates the use high-capacity, complex machine learning models such as the deep neural network approach described here. Leveraging transfer learning and domain adaptation, such as the ensemble method used here, is an important tool to regularize models created from smaller domain-specific corpora with data from external corpora. In the SBD task, the clinical-specific structuring of sentences in our target corpus led us to applying these approaches.

In all experiments, recall was higher than precision, which can be explained by the class weighting structure. Models are penalized much higher for missing a ‘B’ tag than for replacing an ‘I’ with a ‘B’ tag, leading to models being overeager in splitting sentences. Adaptation of models trained on one corpus to another corpus of text show clear but relatively small losses in performance—we can see that the MIMIC trained model has an F1-score approximately 0.06 lower than the FV trained model.

The ensemble method slightly improves the F1 score against the other domain adaptation methods, increasing precision at a slight cost to recall. The ensemble method was the best performing overall with a 0.96 F1 score on B-tags. This F1-score is on par with the 0.957 Cohen’s Kappa inter-rater agreement on the MIMIC data that represents a “ceiling” for performance of SBD algorithms. As shown in the information about our corpora, the FV corpus has different distribution of sentence ‘B’, ‘I’, and ‘O’ tags than the MIMIC corpus, demonstrating that these methods are successful in adapting to a corpus with significant syntactic differences.

There is a loss in performance in the continued training method versus the MIMIC+FV method. In this method, weights may not be able to recover from sub-optimal positions for predicting FV data from the training on MIMIC data. The gradient descent may not be able to find a path from the current position of the weights to the more optimal position of weights found by the FV-only and the MIMIC+FV trained models.

## Conclusions

Our study shows that there are improvements in SBD using deep networks over using traditional classification methods, and that these networks can perform well even against different corpora and against corpora with large proportions of sentences that are not terminated by sentence boundary symbols. We’ve also shown that transfer learning approaches for domain adaptation such as the ensemble model have lower generalization error than combining training sets or continued training.

### Further Work

The generalization performance gains from using a two-network ensemble indicate further exploration into meta-learning and ensemble approaches may be fruitful. Furthermore, usage of this or other transfer learning ensemble methods with general-domain English corpora included as training data for base models remains an unexplored possibility.

The accuracy of automatically detected sentences can have substantial consequences on downstream components in a processing pipeline. These benefits are significant on face but have not been formally quantified, and these effects are a potential target for future research.

## Figures and Tables

**Table 1 –:** Examples of sentences without termination

Text
RECOMMENDATIONS FOR MDs/PROVIDERS TO ORDER:
Recommendations already ordered by Registered Dietitian (RD): Calorie counts reordered
Diet: dysphagia diet level 2 mechanical, thin liquids, magic cup between meals, Nepro between meals
Pt reported his appetite is getting better, he likes the supplements
(+) No chance of pregnancy C-spine cleared: N/A, no H/O Chronic pain,no other significant disability

**Table 2 –:** Inpatient note types per FV batch

Note Type	Number
Progress Note	3
Plan of Care	3
ED Notes	2
8 other note types	1 each

**Table 3 –:** Outpatient note departments per FV batch

Note Type	Number
Family Medicine	5
Internal Medicine	4
Pediatrics	3
Obstetrics and Gynecology	3
Hematology and Oncology	3
Urgent Care	3
Physical Therapy	3
Cardiovascular Disease	3
13 other departments	1 each

**Table 4 –:** Distribution of Tags in MIMIC

Tag	Count	Percentage
B	23,648	7.5%
I	200,272	63.4%
O	91,877	29.1%

**Table 5 –:** Distribution of Tags in FV

Tag	Count	Percentage
B	43,636	10.5%
I	336,018	80.9%
O	35,458	8.5%

**Table 6 –:** Sentence Termination Type

Type	MIMIC	FV
Period	12,698 (53.7%)	13,619 (31.2%)
Exclamation Point	4	19
Question Mark	24 (0.1%)	261 (0.6%)
Semi-colon	48 (0.2%)	10
Colon	4,855 (20.5%)	6,180 (14.2%)
Quotation	4	58 (0.1%)
No symbol	6,018 (25.4%)	23,506 (53.8%)

**Table 7 –:** ‘B’ Tag Accuracy Against FV Hold-out

Method	Precision	Recall	F1
LR-MIMIC	0.511	0.840	0.636
LR-FV	0.650	0.948	0.771
MIMIC	0.829	0.971	0.895
FV	0.923	0.991	0.956
MIMIC+FV	0.919	**0.995**	0.956
MIMIC then FV	0.910	0.992	0.949
Ensemble	**0.933**	0.989	**0.96**
